# Supplementary figures and images for: Genomic screening of allelic and genotypic transmission ratio distortion in horse
Source: PLoS One. 2023 Aug 9;18(8):e0289066. doi: 10.1371/journal.pone.0289066 (PMC10411798; doi:10.1371/journal.pone.0289066)

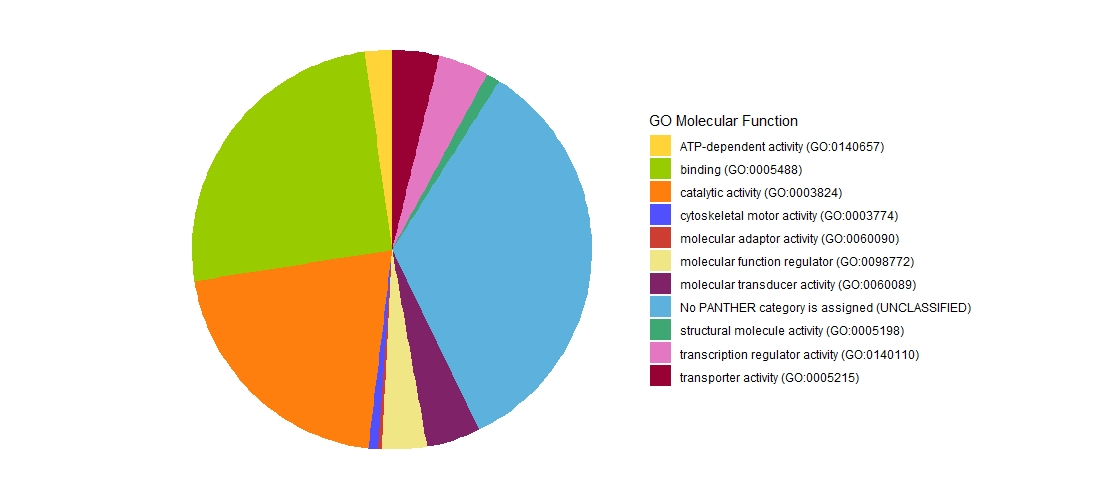

Supplement: S1 Fig — Including A) molecular functions, and B) cellular components. (ZIP) [file pone.0289066.s006.zip › S1A_Fig.tif]

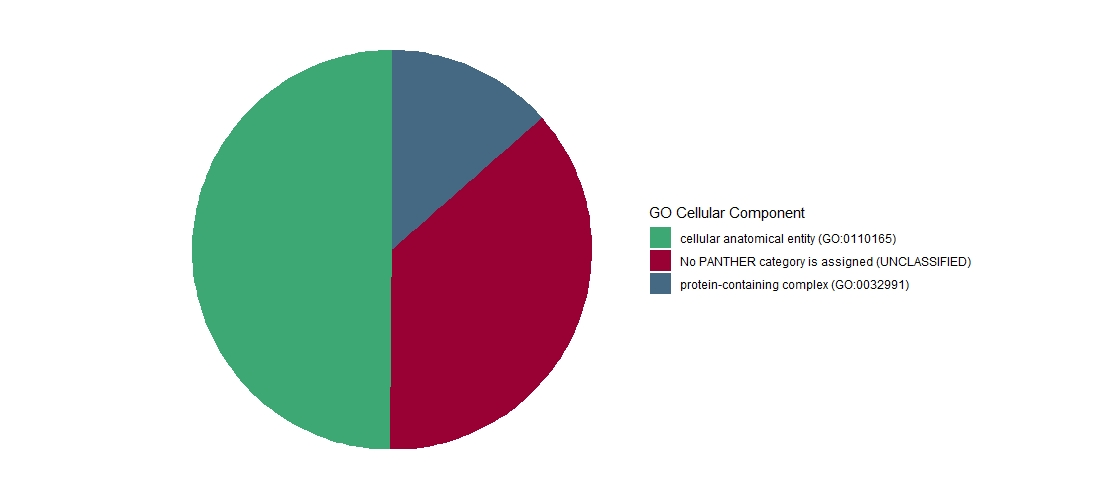

Supplement: S1 Fig — Including A) molecular functions, and B) cellular components. (ZIP) [file pone.0289066.s006.zip › S1B_Fig.tif]

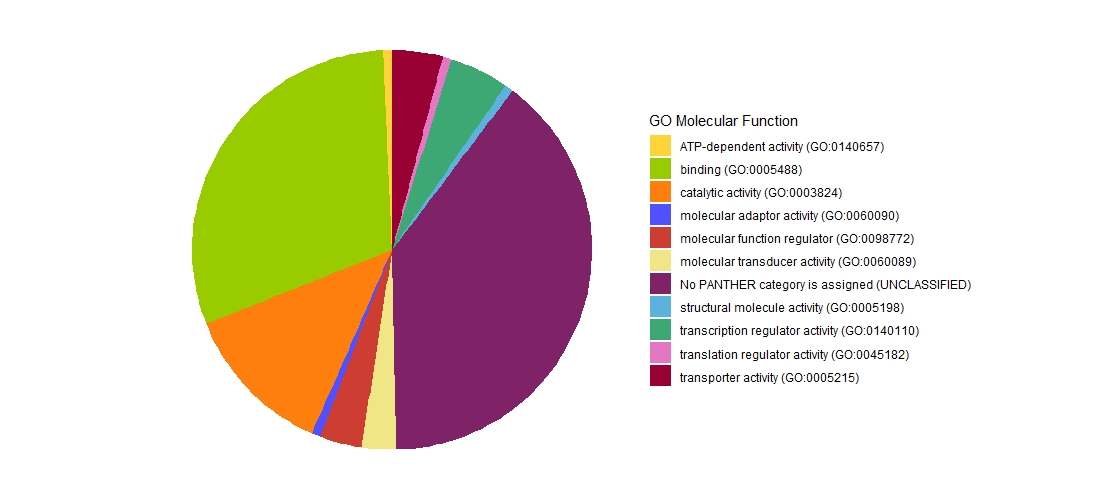

Supplement: S2 Fig — Including A) molecular functions, and B) cellular components. (ZIP) [file pone.0289066.s007.zip › S2A_Fig.tif]
